# Neuron-specific enolase enzymatic activity as a biomarker to discriminate neurologic and orthopedic gait abnormalities in dogs

**DOI:** 10.1093/jvimsj/aalag122

**Published:** 2026-06-24

**Authors:** Amy Huynh, Michael J Byron, Margret I Lenfest, Furkat Mukhtarov, Rubina Yasmin, Roy Cohen, Mark Rishniw, Alexander J Travis, Yael Merbl

**Affiliations:** College of Veterinary Medicine, Department of Clinical Sciences, Cornell University, Ithaca, NY, United States; College of Veterinary Medicine, Department of Clinical Sciences, Cornell University, Ithaca, NY, United States; College of Veterinary Medicine, Department of Clinical Sciences, Cornell University, Ithaca, NY, United States; TETmedical, Inc., 22 Thornwood Drive, Ithaca, NY, United States; TETmedical, Inc., 22 Thornwood Drive, Ithaca, NY, United States; TETmedical, Inc., 22 Thornwood Drive, Ithaca, NY, United States; Cornell University, College of Veterinary Medicine, Baker Institute for Animal Health, Ithaca, NY, United States; College of Veterinary Medicine, Department of Public & Ecosystem Health, Cornell University, Ithaca, NY, United States; College of Veterinary Medicine, Department of Clinical Sciences, Cornell University, Ithaca, NY, United States; TETmedical, Inc., 22 Thornwood Drive, Ithaca, NY, United States; Cornell University, College of Veterinary Medicine, Baker Institute for Animal Health, Ithaca, NY, United States; College of Veterinary Medicine, Department of Public & Ecosystem Health, Cornell University, Ithaca, NY, United States; College of Veterinary Medicine, Department of Clinical Sciences, Cornell University, Ithaca, NY, United States

**Keywords:** biomarker, canine, neuron-specific enolase, neuron-specific enolase activity, neuronal injury

## Abstract

**Background:**

The glycolytic enzyme neuron-specific enolase (NSE) shows high expression in neurons, generating interest as a diagnostic biomarker for neurologic diseases. However, assays measuring NSE protein concentration (NSE-p) have yielded inconsistent diagnostic results.

**Hypothesis/Objectives:**

This was an observational study to investigate NSE enzymatic activity (NSE-a) as a biomarker in dogs to distinguish neurologic vs musculoskeletal causes of gait abnormalities. We hypothesized that NSE-a would be superior to NSE-p for detecting neurologic conditions.

**Animals:**

Client-owned dogs presenting with gait abnormalities were enrolled as: (1) neurology service (*n* = 10), (2) orthopedic surgery and sports medicine and rehabilitation (SMR) (*n* = 21), and (3) control dermatology patients without gait abnormalities (*n* = 3). Inclusion required clinical examination by diplomates or residents of the relevant service and confirmed diagnoses based on radiography, ultrasonography, computed tomography, and magnetic resonance imaging.

**Methods:**

Serum NSE-p was measured using validated commercial ELISA kits for use in dogs. Plasma NSE-a was quantified using an NSE functional activity assay (NSE-FA, TETmedical). A secondary experiment compared the techniques using plasma samples from dogs with neurological gait abnormalities.

**Results:**

Neurologic dogs showed significantly increased plasma NSE-a (median, 0.372; IQR, 0.274-0.407) compared with orthopedic and SMR dogs (median, 0.212; IQR, 0.154-0.259; *P* = .001) and controls (median, 0.218; IQR, 0.15-0.226; *P* = .03). Direct comparison indicated that NSE-p fell below detection thresholds whereas NSE-a remained measurable.

**Conclusions and clinical importance:**

Our results suggest that NSE-a is superior to NSE-p for differentiating neurologic from musculoskeletal gait abnormalities. Future research should explore its potential as a point-of-care biomarker for patients with suspected neurologic conditions.

## Introduction

Gait abnormality is a common clinical complaint in dogs, mostly caused by either a neurologic or musculoskeletal disease.^1^ In veterinary medicine, some types of gait abnormalities are easily diagnosed, whereas others present a diagnostic challenge requiring time, advanced diagnostic testing, and familiarity with functional anatomy to identify the correct cause.^1^ The diagnostic process to find the source of lameness relies, in addition to thorough orthopedic and neurologic examinations, on several diagnostic tests, such as radiography, arthroscopy, computed tomography (CT), and magnetic resonance imaging (MRI) of the spinal cord and peripheral nerves.^2^ These modalities carry substantial financial costs, necessitate general anesthesia, and sometimes delay treatment. Even with extensive imaging assessment, diagnostic findings still may be inconclusive, and a reliable biomarker could improve diagnostic accuracy and clinical decision-making. Many biomarkers have been suggested to identify neurologic and orthopedic conditions, but currently no biomarker can differentiate between these conditions.

Neuron-specific enolase (NSE) is a glycolytic intracellular enzyme that is highly expressed in central and peripheral neurons as well as in neuroendocrine cells with a biological half-life in blood ranging from 24 to 72 h in humans.^3^ Neuronal damage causes the release of NSE into the extracellular space, thereby increasing its concentration in the blood. Many studies have evaluated the changes of NSE protein concentration (NSE-p) in response to known neurologic conditions as well as assessed its potential to serve as a prognostic indicator with variable degrees of success.^4-13^ Enzymatic activity of NSE (NSE-a) has been suggested as an alternative method to measure NSE concentrations.^14-16^ Limited data are available on the diagnostic utility of NSE-a compared to NSE-p in neurologic conditions, and NSE-a has not previously been measured or reported in dogs.

Our aims were to: (1) compare the detection ability of NSE-p concentration as measured by validated ELISA kits designed for use in dogs (using serum and plasma) compared with plasma NSE-a using tethered enzyme technology and (2) to determine whether these biomarkers can predict a neurologic cause of gait abnormalities in dogs. We hypothesized that both NSE-p and NSE-a would be increased in patients with gait abnormalities of a neurologic origin and that NSE-a will be more sensitive in predicting a neurologic cause of altered gait.

## Materials and methods

### Study design

Ours was a prospective observational study conducted at the Cornell University Hospital for Animals. Client-owned dogs presenting with gait abnormalities were enrolled in 2 phases. Dogs admitted to the neurology service (neurologic group), orthopedic surgery or sports medicine and rehabilitation service (orthopedic group), or dermatology service without gait abnormalities (control group) were recruited. Inclusion required clinical examination by a diplomate or resident of the respective service and a confirmed diagnosis based on radiography, ultrasonography, CT, or MRI. Exclusion criteria included witnessed or suspected polytrauma and preexisting concurrent neurologic and orthopedic conditions.

### Data collection

Data collection included signalment (age, sex, neuter status, and breed), neurologic status if applicable, clinical diagnosis, and modality used to obtain the diagnosis. A modified Frankel score (MFS) was used for patients with a neurologic gait abnormality and was defined as: 0 = paraplegic deep pain negative, 1 = paraplegic deep pain positive, 2 = nonambulatory tetra- or paraparetic, 3 = ambulatory tetra- or paraparetic, 4 = spinal hyperesthesia only, or 5 = normal.

### Sample collection and analysis

Blood samples were collected from all dogs in phase 1 (*n* = 34) for measurement of plasma NSE-a using the NSE functional activity assay (NSE-FA; TETmedical Inc., Ithaca, NY). In phase 2 (*n* = 13), the same plasma samples were used to measure both NSE-a and serum NSE protein concentration (NSE-p) using 3 commercially available canine ELISA kits (MyBioSource MBS736549; Biorbyt orb866978; Novatein Biosciences AYQ-E10693) for direct comparison. The ELISA kits were validated for use in dogs using parallelism and spike and recovery testing before analysis.

The primary outcome was plasma NSE-a (NSE-Score) as a discriminator between neurologic and non-neurologic causes of gait abnormality. A secondary outcome was direct comparison of NSE-a with NSE-p in the same plasma samples.

### Statistical analysis

Statistical analyses were performed using GraphPad Prism 10 and Excel. Given the small control group (*n* = 3) and inability to confirm normality, a Kruskal–Wallis test with Dunn’s multiple comparisons was used to compare NSE-a across diagnostic groups. Results are reported as median and IQR. Receiver operating characteristic (ROC) curve analysis was performed to assess diagnostic accuracy. *P* < .05 was considered statistically significant.

Detailed descriptions of case selection, sample collection, assay protocols, and validation procedures are provided in the Appendix.

## Results

### Animals

In the first phase of the study, the neurologic group consisted of 10 dogs, 6 males (5 neutered, 1 intact) and 4 females (all spayed). Breeds included mixed breed dogs (3/10), French Bulldog (2/10), German Shepherd (2/10), and 1 each of Golden Retriever, Beagle, and Pit Bull Terrier. The median age was 7 years (range, 3-15). The orthopedic group consisted of 21 dogs, 12 male (10 neutered, 2 intact) and 9 female (all spayed). Breeds included Labrador Retriever (8/21), mixed-breed dogs (5/21), Golden Retriever (2/21), and 1 each of Beagle, Australian Cattle Dog, Siberian Husky, Boxer, Irish Wolfhound, and Pit Bull Terrier. The median age was 8 years (range, 1-13). The control group consisted of 3 dogs, 2 males (1 neutered and 1 intact) and 1 female (intact). Breeds included mixed-breed dog, Plott Hound, and Bloodhound. The median age was 9 years (range, 4-12). The clinical data for dogs recruited in phase 1 are summarized in Table 1.

**Table 1:** Clinical data of dogs in phase 1. Acute is defined as < 4 days, subacute as 4-14 days, and chronic as > 14 days.

**Subject ID**	**Sample group**	**Breed**	**M/F I/S**	**Age, years**	**Duration of clinical signs**	**Modality of diagnosis**	**Neurological degree of severity**	**Clinical diagnosis**
**376888**	Control	Bloodhound	MI	4			Normal (MFS 5)	Otitis externa
**351634**	Control	Plott Hound	MN	9			Normal (MFS 5)	Pemphigus foliaceous
**243975**	Control	Mixed	FI	12			Normal (MFS 5)	Pemphigus foliaceous
**376036**	Ortho	Golden Retriever	MN	4	Chronic	Ultrasound	Normal (MFS 5)	Left patellar tendinopathy
**376182**	Ortho	Labrador Retriever	MN	2	Chronic	CT	Normal (MFS 5)	Bilateral elbow dysplasia and secondary osteoarthrosis
**284919**	Ortho	Labrador Retriever	FS	10	Chronic	CT	Normal (MFS 5)	Bilateral elbow dysplasia and osteoarthrosis
**366828**	Ortho	Beagle	MN	1	Chronic	CT	Normal (MFS 5)	Bilateral angular limb deformity
**342529**	Ortho	Labrador Retriever	FS	9	Chronic	Radiograph, arthroscopy	Normal (MFS 5)	Right cranial cruciate ligament tear
**328167**	Ortho	Australian Cattle Dog	FS	10	Chronic	Radiograph	Normal (MFS 5)	Right cranial cruciate ligament tear
**222348**	Ortho	Labrador Retriever	FS	13	Chronic	Radiograph	Normal (MFS 5)	Right elbow osteoarthrosis, multifocal intervertebral disc protrusion
**270635**	Ortho	Mixed	MN	6	Chronic	CT	Normal (MFS 5)	Bilateral elbow dysplasia and secondary osteoarthrosis
**262153**	Ortho	Mixed	FS	9	Chronic	CT	Normal (MFS 5)	Bilateral elbow dysplasia and secondary osteoarthrosis
**326695**	Ortho	Golden Retriever	FS	5	Chronic	CT	Normal (MFS 5)	Bilateral elbow osteoarthritis
**250787**	Ortho	Labrador Retriever	FS	9	Chronic	Radiograph	Normal (MFS 5)	Bilateral tarsal osteochondritis dissecans
**365236**	Ortho	Mixed	FS	1	Chronic	Radiograph	Normal (MFS 5)	Bilateral femoral head ostectomy
**352788**	Ortho	Mixed	MN	11	Chronic	Ultrasound	Normal (MFS 5)	Bilateral Achilles tendinopathy
**351511**	Ortho	Labrador Retriever	MN	5	Chronic	CT	Normal (MFS 5)	Bilateral elbow dysplasia and secondary osteoarthrosis
**353644**	Ortho	Labrador Retriever	MI	9	Chronic	Radiograph	Normal (MFS 5)	Left cranial cruciate ligament injury
**376961**	Ortho	Siberian Husky	MN	4	Chronic	Radiograph	Normal (MFS 5)	Hip dysplasia
**352039**	Ortho	Boxer	MN	8	Chronic	Radiograph	Normal (MFS 5)	Hip and elbow osteoarthritis
**282647**	Ortho	Labrador Retriever	MN	5	Chronic	CT, arthroscopy	Normal (MFS 5)	Left elbow dysplasia
**361482**	Ortho	Irish Wolfhound	MI	1	Chronic	CT	Normal (MFS 5)	Bilateral shoulder osteochondritis dessicans
**275890**	Ortho	Mixed	MN	10	Chronic	CT	Normal (MFS 5)	Bilateral elbow dysplasia with osteoarthrosis
**309513**	Ortho	Pitbull	FS	8	Chronic	CT	Normal (MFS 5)	Bilateral elbow dysplasia and tarsal osteoarthrosis
**377232**	Neuro	French Bulldog	MN	3	Acute	MRI	Nonambulatory paraparetic (MFS 2)	Intervertebral disc extrusion at L1-3
**377262**	Neuro	German Shepherd	FS	5	Subacute	MRI	Nonambulatory tetraparetic (MFS 2)	Myelitis of unknown etiology
**377458**	Neuro	Golden Retriever	FS	5	Acute	MRI	Paraplegic deep pain negative (MFS 0)	Acute noncompressive nucleus pulposus extrusion
**377804**	Neuro	Mixed	FS	7	Acute	CT, MRI	Paraplegic deep pain positive (MFS 1)	T12-T13 intervertebral disc extrusion and vertebral luxation
**377788**	Neuro	French Bulldog	MI	7	Acute	MRI	Ambulatory tetraparetic (MFS 3)	Intervertebral disc extrusion at C3-4
**377769**	Neuro	Beagle	MN	15	Acute	MRI	Paraplegic deep pain positive (MFS 1)	Intervertebral disc extrusion at T13-L1
**377854**	Neuro	German Shepherd	FS	11	Acute	MRI	Ambulatory paraparetic (MFS 3)	Intervertebral disc extrusion at T13-L1
**368631**	Neuro	Pitbull	MN	7	Chronic	MRI	Ambulatory tetraparetic (MFS 3)	Intervertebral disc herniation at C6-7
**377502**	Neuro	Mixed	MN	4	Chronic	MRI	Nonambulatory paraparetic (MFS 2)	Extradural mass at L2 causing spinal cord compression
**377924**	Neuro	Mixed	MN	9	Acute	MRI	Nonambulatory paraparetic (MFS 2)	Acute noncompressive nucleus pulposus extrusion at T13-L1

Abbreviations: CT, computed tomography; MFS, modified Frankel score; MRI, magnetic resonance imaging.

In the second phase of the study, the neurologic group consisted of 6 dogs, 3 males (all neutered) and 3 females (all spayed). Breeds included mixed-breed dogs (2/6) and 1 each of French Bulldog, Basset, Beagle, and Boxer. The median age was 4 years (range, 4-6). The control group consisted of 7 dogs, 4 males (all neutered) and 3 females (all spayed). Breeds included mixed-breed dog (3/7), and 1 each of English Bulldog, Miniature Poodle, Bernese Mountain Dog, and Swiss Mountain Dog. The median age was 7 years (range, 2-10). The clinical data for dogs recruited in phase 2 are summarized in Table 2.

**Table 2:** Clinical data of dogs in phase 2.

**Subject ID**	**Sample group**	**Breed**	**M/F I/S**	**Age, years**	**Duration of clinical signs**	**Neurological degree of severity**	**Clinical diagnosis**
**384943**	Neuro	Mixed	MN	4	Acute	Ambulatory paraparesis (MFS 3)	Intervertebral disc extrusion at T12-T13
**385159**	Neuro	Boxer	MN	6	Chronic	Ambulatory paraparesis (MFS 3)	Intramedullary neoplasia at T8-T12
**366140**	Neuro	French Bulldog	MN	4	Acute	Paraplegic deep pain negative (MFS 0)	Intervertebral disc extrusion at T12-T13
**385560**	Neuro	Bassett Hound	FS	5	Acute	Nonambulatory paraparesis (MFS 2)	Intervertebral disc extrusion at L4-L5
**385667**	Neuro	Mixed	FS	4	Subacute	Paraplegic deep pain negative (MFS 0)	Intervertebral disc extrusion at T13-L1
**385680**	Neuro	Beagle	FS	4	Acute	Ambulatory paraparesis (MFS 3)	Intervertebral disc extrusion at T13-L1
**240676**	Control	Miniature Poodle	FS	10	NA	Normal (MFS 5)	Resolved acute kidney injury
**381487**	Control	Mixed	MN	7	NA	Normal (MFS 5)	Periodontal disease
**381126**	Control	English Bulldog	MN	2	NA	Normal (MFS 5)	Atopy
**299531**	Control	Mixed	FS	9	NA	Normal (MFS 5)	Periodontal disease
**382044**	Control	Bernese Mountain Dog	MN	8	NA	Normal (MFS 5)	Hip dysplasia
**385061**	Control	Swiss Mountain Dog	FS	4	NA	Normal (MFS 5)	Laparoscopic ovariectomy
**386785**	Control	Mixed	MN	2	NA	Normal (MFS 5)	Hematochezia

Abbreviation: MFS, modified Frankel score.

### NSE concentration via commercial ELISAs

All 3 commercial assays used were validated for canine samples and were used according to the manufacturer’s instructions. However, they produced inconsistent and unexpected results for quantification of NSE concentration. A parallelism test for the Novatein Biosciences kit resulted in no discernible NSE in any dilution, with optical density values at or below the level of detection for the assay blank. A parallelism test for the Biorbyt kit resulted in similarly low values only slightly above the level of detection for the kit and a coefficient of variation (CV) of 96% (Table 3). Although a parallelism test using the MyBioSource kit did provide results that were within the range of the assay for the first several dilutions, poor linearity was seen in these samples, with a CV of 108% (Table 3).

**Table 3:** Parallelism results from each of the 3 commercially available assays tested.

	**MyBioSource**		**Biorbyt**		**Novatein Bio**	
**Dilution**	**Final calculated concentration (ng/mL)**	**CV**	**Final calculated concentration (ng/mL)**	**CV**	**Final calculated concentration (ng/mL)**	**CV**
**Neat**	32.7	108%	2.5	96%	OOR<	N/A
**1:2**	11.6	4.0	OOR<			
**1:4**	0.78	4.0	OOR<			
**1:8**	OOR<	4.2	OOR<			
**1:16**	OOR<	17.4	OOR<			
**1:32**	OOR<	13.4	OOR<			

Abbreviations: CV, coefficient of variation; N/A, not available; OOR, out of range.

Results shown are the final calculated concentration for each sample in ng/mL (concentration of each sample as calculated by the ELISA kit multiplied by the dilution factor) and the CV (SD of the set of final calculated concentrations divided by the mean of the set, multiplied by 100). Sample concentrations that were below the range of the assay were classified as “Out of range Low” (OOR<).

Results from the spike and recovery tests showed possible poor ELISA compatibility with the sample matrix. The Biorbyt kit showed poor recovery of the NSE analyte in the sample matrix (serum) at low dilutions (Table 4). In addition, the results for this kit showed poor linearity outside of the generally accepted values of 80%-120% (Table 4). The Novatein Bio kit showed poor recovery of the NSE analyte in neat samples and high recovery in the sample matrix at dilutions of 1:2 and higher (Table 4). In addition, linearity values were poor until reaching dilutions of 1:8 or higher. The combination of the recovery and linearity results could suggest the potential for sample matrix interference with the assay at low dilutions, preventing the reading of accurate and reliable concentrations.

**Table 4:** Results from spike and recovery and linearity tests for the Biorbyt and Novatein Bio assays.

	**Biorbyt**				**Novatein Bio**			
**Dilution**	**Expected conc. (ng/mL)**	**Observed conc. (ng/mL)**	**Percent recovery**	**Linearity**	**Expected conc. (ng/mL)**	**Observed conc. (ng/mL)**	**Percent recovery**	**Linearity**
**Neat**	20	2.18	10.9%		2.5	2.05	82%	
**1:2**	10	2.23	22.3%	204%	1.25	1.92	154%	187%
**1:4**	5	1.24	24.8%	111%	0.625	1.27	203%	132%
**1:8**	2.5	0.84	33.6%	136%	0.3125	0.75	241%	119%
**1:16**					0.156	0.38	245%	102%
**1:32**					0.078	0.26	329%	134%

Abbreviation: NSE, neuron-specific enolase.

Three samples were pooled together and spiked with NSE standard analyte at a concentration of either 20 ng/mL (Biorbyt) or 2.5 ng/mL (Novatein Bio), and then serially diluted 1:2 into assay diluent buffer. Percentage recovery was calculated by dividing the observed concentration by the expected concentration, multiplied by 100. Linearity was calculated by dividing the final concentration (with dilution factor accounted for) of each sample by the final concentration of the previous sample in the dilution series, multiplied by 100.

### Measuring NSE enzymatic activity using the NSE-FA assay

**Figure 1:**
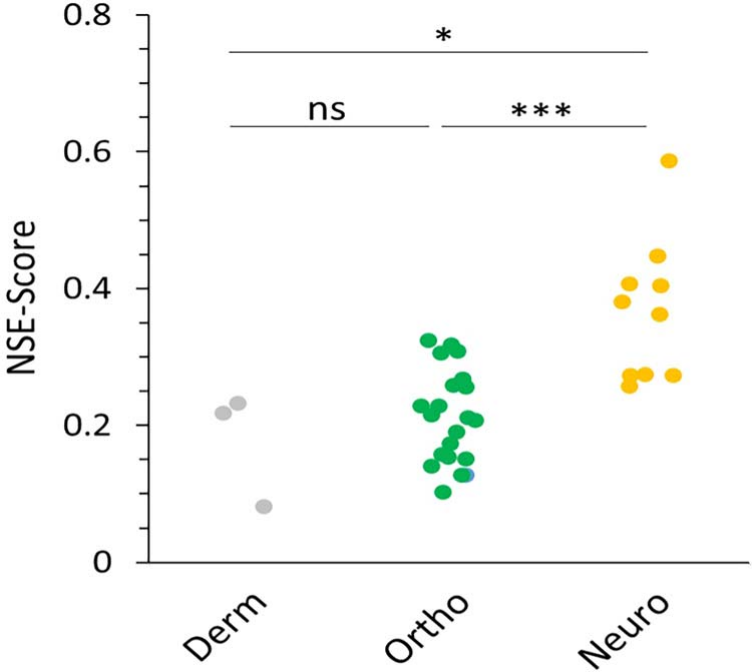
Relative NSE-a differentiates between neurologic and non-neurologic causes of gait abnormalities. Phase 1 patients were divided into 3 groups based on their condition or diagnosis: dermatologic (*n* = 3), orthopedic (*n* = 21), and neurologic (*n* = 10). NSE-score was calculated from luminescence signal slopes: NSE-score = (test slope − negative slope)/(positive slope − negative slope). The neurologic group had a significantly higher NSE-score than both the dermatologic group (*P* = .03) and the orthopedic group (*P* = .001) (adjusted for multiple testing), indicating higher NSE activity in the sample. Abbreviations: NSE = neuron-specific enolase; NSE-a = neuron-specific enolase enzymatic activity.

In phase 1 of the study, we compared NSE-a levels concentrations among the 3 different study groups. We found significant increases in NSE-a concentrations (median, IQR) in the neurologic group (0.372; 0.274-0.407; *n* = 10) compared with the orthopedic (0.212; 0.154-0.259; *n* = 21; *P* = .001) or dermatologic (0.218; 0.15-0.226; *n* = 3; *P* = .03) groups (Figure 1). Of the patients tested in phase 1, 3 dermatologic, 1 orthopedic, and 1 neurologic sample were excluded from analysis because of severe hemolysis (level > 4 according to the Centers for Disease Control [CDC] hemolysis reference palette). One patient from the orthopedic group also was excluded after a later diagnosis of a brain tumor.

**Figure 2:**
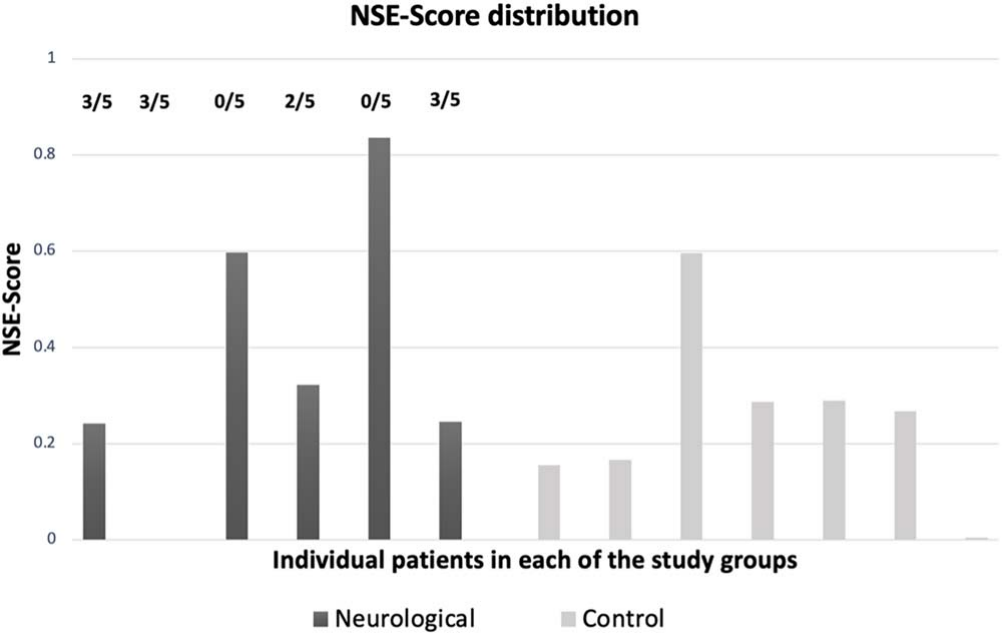
Relative NSE-a differentiated between neurologic and control patients in phase 2 of the study. Patients were divided into 2 groups based on their condition or diagnosis: neurologic (*n* = 6) vs. non-neurologic patients, and neurologic (*n* = 6). NSE-score was calculated from luminescence signal slopes: NSE-score = (test slope − negative slope)/(positive slope − negative slope). Severity of neurologic deficits is listed above each neurologic patient based on the modified Frankel score (0-5) as previously described in the text. NSE-score was increased in neurologic patients with more severe neurologic grading, indicating higher NSE activity. Abbreviations: NSE = neuron-specific enolase; NSE-a = neuron-specific enolase enzymatic activity.

In phase 2, samples from a separate cohort of dogs with neurologic causes of gait abnormality and controls were assessed, using the same plasma samples for both NSE-a and NSE-p. We found that NSE-a was measurable in canine plasma for most dogs in both the control and study groups (Figure 2) whereas NSE-p was not detected in any of the samples. Interestingly, the dogs with the highest MFS severity score had higher NSE-a concentrations.

**Figure 3:**
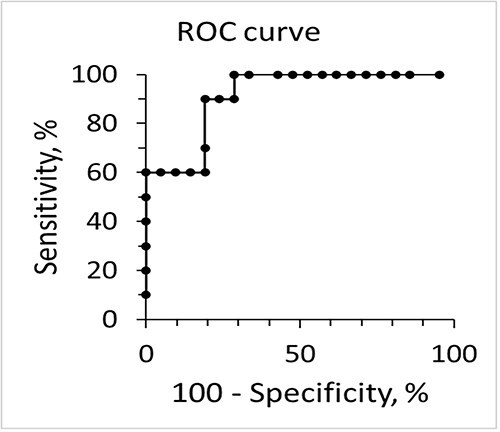
ROC curve for canine NSE in distinguishing neurologic from orthopedic patients. The AUC was 0.914 (95% CI, 0.816-1.000; *P* = .0002), indicating excellent diagnostic discrimination. Each point represents a potential threshold value for NSE concentration. Abbreviations: AUC = area under the curve; NSE = neuron-specific enolase; ROC = receiver operating characteristic.

The ROC analysis of phase 1 data demonstrated excellent diagnostic accuracy for NSE-a in distinguishing neurologic from non-neurologic cases (AUC, 0.914; 95% CI, 0.816-1.000; *P* = .0002). At an NSE-score threshold of 0.257, the assay achieved 100% sensitivity and 66.7% specificity (Figure 3).

## Discussion

Our major findings were that NSE-a is measurable in canine plasma whereas NSE-p was not consistently detected in any samples. Neuron-specific enolase enzymatic activity was also significantly increased in dogs with a neurologic cause of gait abnormalities as compared with orthopedic causes or controls without gait abnormalities. These results supported rejection of our hypothesis that both NSE-p and NSE-a would be increased in patients with a neurologic cause of gait abnormality, but supported the hypothesis that NSE-a would be more sensitive in predicting a neurologic cause of altered gait. Additional clinical research on NSE-a’s diagnostic potential is warranted because the commercial ELISA tests did not perform with the necessary rigor or levels of detection for clinical utility.

Increased serum NSE-p has been reported in response to a variety of neurologic conditions in humans such as spinal cord injury, traumatic brain injury, infarcts, seizures, and encephalitis.^4-10^ Furthermore, the severity of serum NSE-p increase correlated with the severity of spinal cord injury or traumatic brain injury, suggesting its potential utility as a prognostic indicator in these cohorts of patients.^8^^,^^9^ Increased serum NSE-p also has been demonstrated in dogs with autoimmune or infectious encephalitis and lysosomal storage disorders, and the severity of increase correlated with clinical severity in the latter disease.^10^^,^^17^^,^^18^ More recently, serum NSE-p was evaluated as a prognostic indicator in dogs with traumatic brain injury without clinically relevant results, although serum samples were stored up to 5 years.^19^ Prolonged serum storage previously has been reported to decrease NSE-p because of protein degradation.^20^ Moreover, all but 1 study utilized ELISAs designed for humans to measure serum NSE-p in dogs rather than using an assay validated for canine NSE. This approach can lead to inaccurate serum NSE concentrations because of differences in affinity between canine NSE antigen and human NSE antibody. We aimed to address these gaps by measuring NSE-p within 3 weeks of sample collection and utilizing ELISA kits designed for dogs.

In our study, we tested 3 different NSE ELISAs reported to be validated for dogs with repeatedly inconsistent results. Parallelism tests using pooled samples of expected high concentrations returned lower than expected concentrations when undiluted, and serial dilutions did not show a similar pattern to the standard curve, suggesting a difference in reactivity with the ELISA kit between the native NSE protein in each sample and the NSE protein used for the standard curve. In addition, spike and recovery tests suggested a high likelihood of matrix interference. In this scenario, elements of the canine serum matrix can interfere with antibody binding, which can explain the low percentage recovery of the Biorbyt and the Novatein Bio kits seen in the neat and low dilution samples. As the sample is diluted out further in the assay diluent buffer, the percentage of the interfering substance is decreased, lowering this effect. Because the percentage recovery for the Novatein Bio kit is very high at high dilutions, this finding may suggest that there are concentrations of endogenous NSE-p in our serum samples that are not being detected at low dilutions because of a matrix effect. Also, for the Novatein Bio kit, our linearity of dilution results suggests that assay results may not be accurate unless the sample is run at a dilution of 1:8 or higher. However, endogenous serum NSE-p concentration may not be high enough to fall within the range of the assay at higher dilutions. A previously available version of the MyBioSource Canine NSE ELISA kit had a detection range of 1-5000 pg/mL, and 1 study was able to use this kit to successfully measure concentrations of NSE-p in dogs.^19^ However, the kit currently available for purchase from MyBioSource is much less sensitive with a detection range of 5-100 ng/mL. The Novatein Bio kit has a higher sensitivity than any other commercial ELISAs currently available, with a low-end range of 0.078 ng/mL. However, it appears that even with this kit, endogenous concentrations of NSE-p may be too low to quantify because of matrix interference, and another more sensitive method is needed for determining the presence of NSE-p in canine serum. The low concentrations below detection in our study also may be attributed to the different nature of injury in brain vs spinal cord tissue. Focal spinal cord injury may cause less neuronal damage compared with brain injury, and presumably very low NSE-p concentrations that were not detected by available NSE ELISA kits designed for dogs.

Measurement of NSE-a is an alternative method of assessing NSE activity and has been demonstrated to be increased in murine models of stroke and traumatic brain injury and in humans with Alzheimer’s disease and neurologic disease in general.^15^^,^^16^^,^^21^ Neuron-specific enolase enzyme activity also appears to be a more sensitive biomarker for neuronal injury with peak NSE-a occurring at 2-3 h postinjury compared with NSE-p, which typically peaks at 6 h postinjury.^15^^,^^21-24^ Measurement of NSE-a also can be achieved rapidly, within 10 min, compared with NSE-p ELISA, which takes approximately 3 h.^25^ The short half-life of NSE activity should enable the NSE-FA to distinguish between NSE molecules newly released from an acute injury vs those that already might be present in the circulation. We assessed the utility of NSE-a in differentiating patients with neurologic disease from orthopedic and control groups as well as to assess the sensitivity of plasma NSE-a compared with NSE-p in the same cohort.

Results showed that serum NSE-a was consistently measurable in all samples unlike NSE-p. Moreover, NSE-a could differentiate between patients with a neurologic condition compared with those with an orthopedic condition (*P* = .001) and compared with the control group (*P* = .03). These findings align with the expected increase in plasma NSE activity in dogs with ongoing neurologic disease. Interestingly, within our cohort, NSE-a could predict a neurologic condition (brain tumor) for a patient with a known orthopedic condition 4 months before the onset of any neurologic signs. This patient was included in the data collection but later was excluded during data analysis because it was unknown if the brain tumor was present at the time of sample collection, but the patient’s NSE-a score was 0.439, compared with an NSE-a median score of 0.372 for the group with neurologic conditions and 0.212 for the group with orthopedic conditions. In addition, NSE-a scores were higher in patients with more severe neurologic grading (Figure 2), suggesting that NSE-a, like NSE-p, may predict the severity of neuronal injury. As a preliminary study, our data lack the statistical power to further assess whether NSE-a can predict neurologic severity, and future studies should investigate this relationship. Overall, NSE-a appears to be a reliable biomarker to identify neurologic conditions, which in turn can facilitate a rapid and accurate diagnosis, as well as allow for earlier initiation of appropriate treatment.

Our study had some limitations. First, because we initially considered NSE-p as the main biomarker, serum samples were originally used to try to validate the commercial kits. However, because of an unexpected lack of sensitivity with the commercial NSE-p ELISA kits, we decided to collect plasma samples in phase 1 of the study to assess NSE-a as a biomarker. Therefore, samples from phase 1 of the study were used to measure NSE-a alone. The second recruitment of clinical cases (phase 2) was meant to allow a direct comparison between the 2 methods, and we aimed to use the same samples to measure both NSE-p and NSE-a concentrations in the same cohort for a direct comparison. For this reason, in phase 2, plasma was used for the comparison, and not serum. However, because the ELISA kits used in our study are compatible with both biological fluids, this difference does not alter our conclusions.

Another limitation is that different services made independent determinations of the neurologic and orthopedic status of a patient rather than having the same evaluators assessing these patients. Subtle abnormalities may have been missed. In addition, the 3 dermatologic patients used as a control group were presumed not to suffer from neurologic or orthopedic disease, but specific tests were not conducted to support this assumption. Similarly, for patients with a neurologic condition, diagnostic tests to rule out a concurrent orthopedic condition were not performed if there was no indication to pursue additional tests if the orthopedic examination (a routine part of the evaluation for neurologic patients) was normal.

We assessed the utility of NSE-p and NSE-a as biomarkers in identifying neurologic abnormalities in cohorts with clear orthopedic or neurologic abnormalities and excluded those with concurrent conditions. In many clinical scenarios, however, thorough orthopedic and neurologic examinations already may be sufficient to differentiate the cause of a gait abnormality without the need to utilize a biomarker. Despite this limitation, NSE-a still has potential clinical value in cases in which the examinations and diagnostics alone are not sufficient to arrive at a diagnosis. For example, a case with concurrent diseases in which the clinical question is which of the conditions is the primary driver for the presenting clinical signs, or in scenarios in which the clinician is trying to differentiate between nerve root signature (a gait abnormality secondary to nerve compression) and lameness (a gait abnormality secondary to orthopedic conditions). Future prospective studies should recruit these specific populations of patients to assess the utility of NSE-a in predicting neurologic conditions.

Because ours was a novel study of NSE-a in dogs, we could not provide a priori assessment. It is nonetheless noteworthy that the NSE-scores for the control group were all below those of neurologic cases. While continuing to optimize the NSE-a assay for use in dogs, we envision that future studies will include a larger sample size to assess the specificity and sensitivity of NSE-a to differentiate the causes of gait abnormalities as well as assess NSE-a as a potential biomarker to screen for neurologic injury.

## Off-label antimicrobial declaration

The authors declare no off-label use of antimicrobials.

## Institutional animal care and use committee or other approval declaration

Samples were obtained with written consent from the pet owners and approved by the Cornell Institutional Animal Care and Use Committee, protocol numbers 2022-0134 and 2023-0084.

## Appendix: Detailed materials and methods

### Study design and case selection

In our prospective observational study, dogs were recruited from the Cornell University Hospital for Animals in 2 phases. Samples from each phase were collected over a 1-month period. In phase 1 of the study, dogs were recruited based on clinical signs indicating obvious gait abnormalities at admission, admitted to either: (1) the neurology service, (2) the orthopedic service before surgery, or (3) the sports and medicine rehabilitation (SMR) service for management of degenerative orthopedic conditions. To meet the inclusion criteria, dogs needed to have a clinical diagnosis of the source of the gait abnormalities based on the orthopedic or neurologic examinations or both by either a diplomate or a resident in the respective services, and supporting radiographs, ultrasonography, or computed tomography (CT) to confirm the cause of orthopedic lameness and CT or magnetic resonance imaging (MRI) to confirm a neurologic cause of gait abnormalities. Dogs that were recruited from the orthopedic and SMR services with a confirmed cause of gait abnormality were grouped together under the term: orthopedic group. Several dogs admitted to the dermatology service served as a control group if there was no history of orthopedic or neurologic disease reported by the owners and no lameness or neurologic deficits were noted on physical examination.

In phase 2 of the study, the goal was to measure both NSE-p and NSE-a in the same sample for direct comparison of the sensitivity of the tests. Case selection in phase 2 therefore focused on recruiting patients with neurologic causes of gait abnormality so that a direct comparison could be made, and dogs presented to other services within the small animal hospital served as controls. Animals were not age or sex matched. Case selection exclusion criteria in both phases included dogs with witnessed or suspected polytrauma and those with preexisting concurrent neurologic and orthopedic conditions.

### Sample collections and analysis

At the onset of the study, canine serum samples were utilized to run initial validation tests of the commercial ELISAs for NSE-p. In phase 1 of the study, NSE-a was measured in each plasma sample (*n* = 34). In phase 2, plasma samples were collected to assess both NSE-p and NSE-a for a direct comparison (*n* = 13). The NSE-p ELISAs utilized both serum and plasma samples, whereas NSE-FA required plasma samples only.

#### NSE-p ELISA blood sample collection

Written consent was obtained from the pet owners for use of clinical samples for future research, which was approved by the Cornell Institutional Animal Care and Use Committee (IACUC protocol # 2022-0134). The degree of hemolysis was estimated visually for each sample according to the CDC hemolysis reference palette because hemolysis is a criterion for sample rejection for NSE-p. Samples that were classified as having a hemolysis score of ≥ 3, indicating moderate to severe hemolysis, were excluded from the analysis. Blood samples were allowed to clot for 30 min at room temperature and then centrifuged at 1000 × *g* for 10 min. Serum then was collected, aliquoted, and stored at −80°C until assayed.

#### NSE-a assay blood sample collection

Blood samples for NSE-a analysis were collected either specifically for this purpose or were collected from clinical samples for future research. The former was approved by the Cornell Institutional Animal Care and Use Committee (IACUC protocol # 2023-0084), and the latter was approved by IACUC protocol #2022-0134. Samples with a hemolysis score of ≥ 4, indicating severe hemolysis, were excluded from the analysis. Two milliliters of blood were drawn into 4 mL sodium heparin vacutainer tubes (Becton Dickinson, NJ, USA) and kept at room temperature. Plasma was separated within 35 min of collection by centrifugation at 1000 × *g* for 10 min at room temperature.

### Serum NSE-p measurement using commercial canine NSE ELISA

Serum NSE-p concentrations were measured using several commercially available ELISA kits for canine NSE. Canine NSE ELISA kits were purchased from 3 different manufacturers (MyBioSource MBS736549; Biorbyt orb866978; Novatein Biosciences AYQ-E10693), with all 3 kits stating that serum and plasma are suitable and comparable samples for the assay. The detection range for each assay was as follows: MBS736549 (MyBioSource, San Diego, CA), 5-100 ng/mL; orb866978 (Biorbyt, Durham, NC), 0.63-40 ng/mL; and AYQ-E10693 (Novatein Bio, Woburn, MA), 0.078-5 ng/mL. All kits were run according to the manufacturer’s instructions. Parallelism and spike and recovery tests were conducted to validate the assays for use in our laboratory. For parallelism experiments, 3 serum samples of expected high concentration were pooled together and then serially diluted 1:2 in respective assay diluent buffer out to 1:32. Optical density values were used to compare differences in binding affinity between the standard analyte and the endogenous analyte, and coefficient of variation (CV) values were calculated by dividing the SD by the mean of the tested sample concentrations while factoring in the dilution factor. Assays displaying good parallelism should provide similar levels of detection of the endogenous analyte at different dilutions. For spike and recovery experiments, 3 samples were pooled together and spiked with NSE standard analyte at a concentration of either 20 ng/mL (Biorbyt) or 2.5 ng/mL (Novatein Bio) and then serially diluted 1:2 into assay diluent buffer out to 1:32. Percentage recovery values for each dilution were calculated to determine linearity.

### Plasma NSE-a measurement using NSE-FA assay

The functional enzymatic activity of NSE (NSE-a) was measured in canine plasma using the NSE-FA assay (TETmedical Inc, Ithaca, NY). The test was performed in 8-well strips, in which each well contained freeze-dried reagents, including pyruvate kinase and firefly luciferase, tethered to silica nanoparticles, with active enolase also freeze-dried in the high-reference wells. Additional reagents included 2-phosphoglycerate (2PG), adenosine diphosphate, and luciferin as previously reported.^21^

To activate the assay, 40 μL of fresh plasma was added to the freeze-dried reagents in the 8-well strip. Luminescence signal coming from the coupled enzymatic activity was measured using a BioTek Synergy H1 plate reader. Luminescence from each well was recorded every 6 s for 10 min, and the slope of the linear luminescence increase within the first 3 min was calculated for each well. The following reference wells were used to allow calculation of NSE’s activity:

- Lower-range reference wells (LRF): Strip wells that lacked 2PG.
- Test (T): Strip wells with 2PG.
- High-range reference wells (HRF): included known amounts of enolase and 2PG.
- The NSE-a score was calculated as follows: (T-LRF)/(HRF-LRF)
- Each sample was tested using a single NSE-FA strip that included triplicate HRF, triplicate T, and duplicate LRF wells. Slopes from each strip were averaged for each triplicate or duplicate data set.

### Statistical analysis

Statistical analyses were performed in Excel or GraphPad Prism 10. Because the dermatology group was small (*n* = 3) and normality could not be confirmed, we used a Kruskal–Wallis test to compare NSE-a across diagnostic groups, followed by Dunn’s multiple comparisons test. Where relevant, results are reported as median, IQR. *P* < .05 was considered statistically significant, and statistical significance in figures is provided as * < .05, ** < .01, *** < .001.

## Abbreviations

2PG: 2-phosphoglycerate
CT: computed tomography
CV: coefficient of variation
GAB: gait abnormalities
HRF: high-range reference wells
LRF: lower-range reference wells
MFS: modified Frankel score
MRI: magnetic resonance imaging
NSE: neuron-specific enolase
NSE-FA: neuron-specific enolase functional activity assay
NSE-p: neuron-specific enolase protein concentration
NSE-a: neuron-specific enolase enzymatic activity
SMR: sports medicine and rehabilitation
T: test
